# Wnt4 is not sufficient to induce lobuloalveolar mammary development

**DOI:** 10.1186/1471-213X-9-55

**Published:** 2009-10-30

**Authors:** Young Chul Kim, Rod J Clark, Francisco Pelegri, Caroline M Alexander

**Affiliations:** 1 McArdle Laboratory for Cancer Research, School of Medicine and Public Health, University of Wisconsin-Madison, Wisconsin, USA; 2 Department of Genetics, University of Wisconsin-Madison, Wisconsin, USA

## Abstract

**Background:**

Brisken et al (2000) showed that Wnt4 null mammary glands were deficient in early lobuloalveolar mammary outgrowth during pregnancy, and implicated Wnt4 as an effector for the progesterone-induced mammary growth program. Though ectopic Wnt1 signaling is known to be mitogenic and oncogenic, no endogenously expressed Wnt ligands have ever been directly implicated in mammary growth and morphogenesis. Therefore, we generated conditional transgenic mice to test whether Wnt4 can stimulate mammary epithelial cell growth.

**Results:**

We found that despite pregnancy-associated expression levels of Wnt4, mammary glands did not display the side-branching typical of early pregnancy. Control experiments designed to test the Wnt4 construct in zebrafish reproduced other studies that demonstrated Wnt4-specific phenotypes distinct from Wnt1-induced phenotypes. Indeed, using qPCR-based array analyses, we found that a specific transcriptional target of Wnt4, namely Wnt16, was induced in Wnt4-expressing transgenic glands, to levels equivalent to that of early pregnant glands.

**Conclusion:**

Taken together, we propose that Wnt4 is necessary, but not sufficient, to induce side-branch development.

## Background

Wnt signaling has been implicated in mammary gland development [1], particularly during the specification of mammary placodes from ectoderm (revealed by LEF1-deficient, and K5-dkk1 transgenic glands) [2-4]. Our data have shown that LRP-5, one of two canonical Wnt signaling receptors, is required for ductal stem cell maintenance [5]. Since several Wnt family genes are differentially expressed in the virgin, pregnant, and lactating mammary gland, some of them regulated by ovarian hormones, it would not be surprising if these Wnt proteins have other, as yet unidentified functions [6,7].

During pregnancy, mammary glands undergo substantial changes induced by steroid and peptide hormones. Progesterone signaling is predominantly responsible for the growth and differentiation of the lobuloalveolar lineage that is characteristic of early pregnancy [8]. Brisken et al (2000) concluded that Wnt4 expression was important to side-branching and lobuloalveolar development during pregnancy, and was regulated by progesterone/PR signaling. Thus, *Wnt4 *null epithelium failed to form side-branches and lobuloalveoli in transplanted mammary fat pads during early pregnancy (12 days of pregnancy). Early developmental defects were overcome in late stage of pregnancy [9]. In addition, Wnt4 expression was induced in vitro in cultured mammary epithelial cells by estrogen and progesterone, and also in vivo in a progesterone-dependent manner during the estrus cycle [9,10]. Thus Wnt4 is considered a downstream mediator of progesterone/progesterone receptor (PR)-induced signaling.

Wnt4 is key to several developmental processes. Wnt4 null mice have no kidneys, due to early failure of the nephric mesenchymal-epithelial transition [11]. Wnt4 is also key to establishing gonadal identity in females [12]. Decidualization (uterine stromal differentiation) is induced by progesterone/BMP2, and requires Wnt4 [13]. It is active during motorneuron specification (together with Wnt5a and-b) [14].

The molecular mechanism by which Wnt4 induces proliferation and morphogenesis of mammary epithelial cells has not been investigated. Therefore, for the present study, we developed bi-transgenic mice that express Wnt4 in mammary epithelial cells in response to tetracycline. By mating tet-operator driven Wnt4 transgenic mice with mice expressing reverse tetracycline trans-activator (rtTA) under the control of the mouse mammary tumor virus (MMTV)-LTR promoter, we were able to induce expression of transgene in mammary glands by doxycycline administration. Unexpectedly (perhaps), ectopic Wnt4 expression did not induce hyper side-branching and lobuloalveolar development in virgin mammary glands. This was despite clear evidence that Wnt16, a Wnt4 transactivation target, was induced in response to tetracycline. Thus, we propose that Wnt4 is required but not sufficient for early lobuloalveolar side-branch development.

## Results

### Construction of tetracycline-inducible Wnt4 transgene cassette and generation of doxycycline-inducible Wnt4 transgenic mice

**Figure 1:**
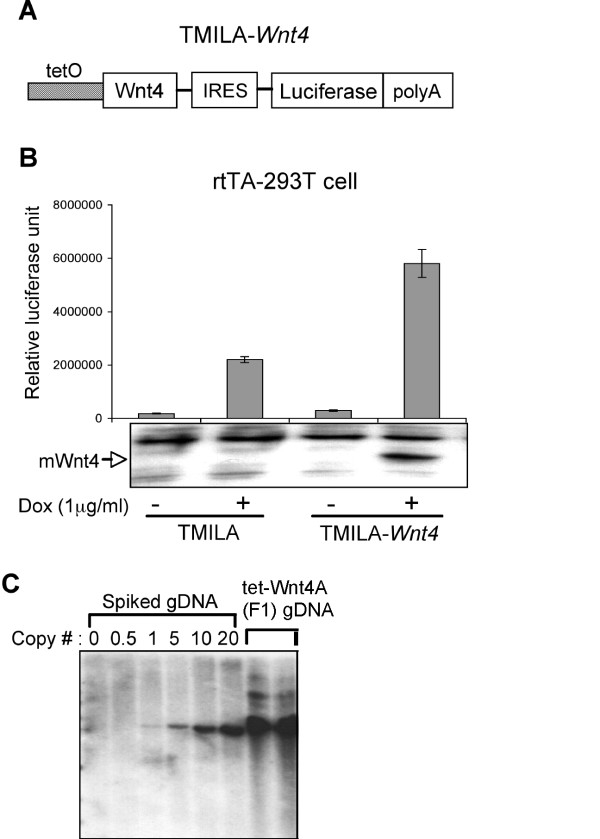
**(A) Diagram of the tetracyline-inducible Wnt4 transgene cassette**. (more details are given in the results section) (B) Expression of Wnt4 and the luciferase reporter is regulated by doxycycline. 293T cells stably expressing rtTA were transfected with either TMILA-Wnt4 vector or parental TMILA vector. Transfected cells were incubated for 24 hrs with or without doxycycline (1 μg/ml), and cell lysates were prepared to measure luciferase activity. Expression of Wnt4 protein was assessed through Western blot analysis using a mouse Wnt4-specific antibody. (C) Southern blot analysis of tet-Wnt4 transgenic mice. Genomic DNA from a non-transgenic mouse was used as a negative control and spiked with TMILA-Wnt4 vector fragment to determine the approximate copy number of the integrated transgene (probes and digestion as described in Methods).

To construct a doxycycline-inducible Wnt4 transgene cassette (TMILA-Wnt4), mouse Wnt4 cDNA was subcloned downstream of tet operator sequences (Fig. 1A), and a minimal CMV promoter into the first cistron of a bicistronic construct. Translation of a luciferase reporter gene via an internal ribosome entry site (IRES) can be used as a surrogate marker for transgene expression [15]. To test whether Wnt4 and luciferase expression was regulated by doxycycline, the TMILA-Wnt4 plasmid was transfected into rtTA-expressing 293T (rtTA-293T) cells, and lysates tested for luciferase activity and Wnt4 protein level (Fig. 1B). This construct showed very low basal levels of both, that were highly induced by the administration of doxycycline (1 μg/ml).

Two independent founder transgenic lines were made by pronuclear injection of the doxycycline-inducible Wnt4 expression cassette (tet-Wnt4-IRES-luciferase) that stably transmitted the transgene to the next generation (tet-Wnt4A and tet-Wnt4B line). Southern blot analysis showed that both lines have >20 copies of transgene (Fig. 1C and tet-Wnt4B data not shown).

### Wnt4 expression is specifically induced in tet-Wnt4/MMTV-rtTA mammary glands by doxycycline-administration

**Figure 2:**
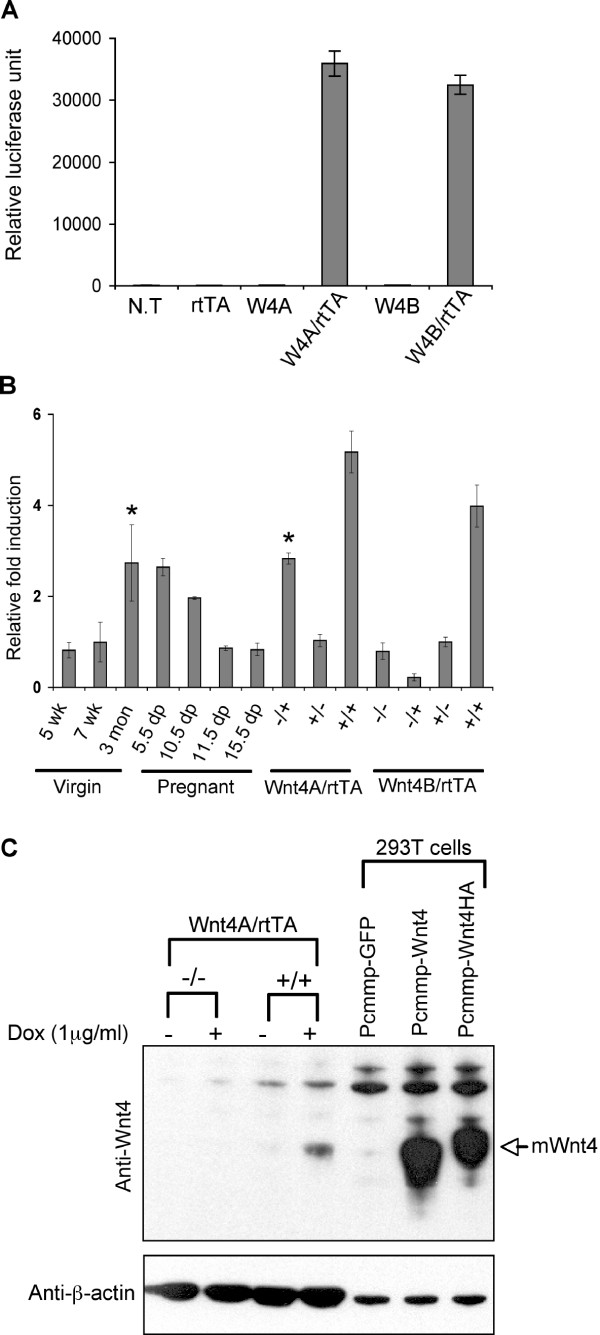
**Characterization of doxycycline-inducible Wnt4 transgenic mice**. (A) Luciferase reporter gene expression in tet-Wnt4/MMTV-rtTA transgenic mammary glands. To express Wnt4 specifically in mammary glands, tet-Wnt4A and B mice were mated with transgenic mice that express rtTA under the control of MMTV-LTR promoter (MMTV-rtTA). The subsequent virgin female littermates (3 months old) were administered doxycycline containing water (2 mg/ml) for 1 month. Mammary gland lysates were prepared, and the luciferase activity of 10 μg of total protein lysate was measured. N.T, non-transgenic; rtTA, MMTV-rtTA; W4A, tet-Wnt4A strain; W4B, independent strain tet-Wnt4B; bitransgenics are shown as W4A or B/rtTA. (B) Induction of Wnt4 mRNA expression in mammary glands in doxycycline-administered mice. Wnt4 mRNA level was quantified by real time quantitative PCR (see methods) using total RNAs from mammary glands of mice administered doxycycline for 1 month. The fold induction is indicated with respect to control glands (from 7 week old mice). For mRNA preparations from non, single and bi-transgenic glands, plus designations indicate the presence of the transgene, and the order is shown as follows, Wnt4A/rtTA. Data marked with an asterix were samples from mice in estrus. (C) Induction of Wnt4 protein expression in doxycycline-treated bitransgenic mammary epithelial cells. Mammary epithelial cells were prepared from non-transgenic (-/-) and bitransgenic (+/+) glands and cultured for 2 days. Cells were treated with doxycycline (1 μg/ml) for another 24 hrs and lysates were prepared for Western blot analysis. Lysates from expression vector-transfected 293T cells were used as positive controls.

In order to express Wnt4 specifically in mammary gland, tet-Wnt4A and B male mice were mated with MMTV-rtTA female mice [16]. Virgin 3~5 month old bitransgenic (tet-Wnt4/MMTV-rtTA) female mice and non- or single-transgenic female littermates were administered doxycyline-water (2 mg/ml) for one month, and luciferase activity in mammary tissue lysates was measured (Fig. 2A). Luciferase reporter gene expression was induced in tet-Wnt4A/MMTV-rtTA or tet-Wnt4B/MMTV-rtTA mammary glands, but not in non-transgenic, tet-Wnt4A, tet-Wnt4B, or MMTV-rtTA mammary glands.

To measure Wnt4 expression directly in doxycyline-treated mammary glands, we measured Wnt4 mRNA level by real time quantitative PCR (qPCR, Fig. 2B), and compared it with normal endogenous expression levels. Consistent with previous reports [6,7,10], Wnt4 mRNA was detected in juvenile and adult virgin mammary glands, and the expression level reached a peak during early pregnancy (5.5~10.5 days postcoitus (d.p.)) declining (relatively) during mid and late pregnancy. In both doxycycline-administered tet-Wnt4A/MMTV-rtTA and tet-Wnt4B/MMTV-rtTA mammary glands, Wnt4 mRNA level was higher than early pregnant mammary glands. Wnt4 mRNA was induced to early pregnant levels in mice in estrus (see samples marked with *), consistent with a previous report of Wnt4 mRNA induction after hormonal exposure [10].

In order to assess induction of Wnt4 protein expression, primary mammary epithelial cells were prepared from tet-Wnt4A/MMTV-rtTA female mice or non-transgenic female littermates and cultured in the presence or absence of doxycycline. Wnt4 protein was only detected in doxycycline-treated bitransgenic mammary epithelial cells (Fig. 2C). Taken together, these data show that the expression of this Wnt4 transgene is effectively induced by doxycyline.

### Ectopic Wnt4 expression is not sufficient to induce hyper side-branching

**Figure 3:**
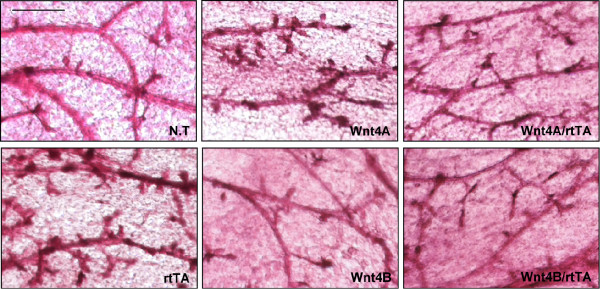
**Ectopic Wnt4 expression is not sufficient to induce hyper side-branching**. To assess side-branching development, inguinal #4 mammary glands were dissected out from the above doxycyline-treated female mice (treated with doxycycline for 1 month at 3 months of age) and stained with Carmine solution. Scale bar = 1 mm.

To assess whether ectopic Wnt4 expression induced side branching and lobuloalveolar growth, we examined whole mount preparations of doxycyline-administered mammary glands (Fig. 3). Surprisingly, neither tet-Wnt4A/MMTV-rtTA nor tet-Wnt4B/MMTV-rtTA mammary glands were significantly different from controls. Control glands that displayed relatively more side-branches and lobuloalveolar development (for instance, the MMTV-rtTA mammary gland shown in Fig. 3) were subsequently shown to have estrus-associated levels of Wnt 4 mRNA.

Shorter or longer periods of doxycycline administration (6 days and 3 months, respectively), or constant administration from embryonic stages *in utero *or from 3 weeks old to 3 months old also did not induce side-branch development in bitransgenic mammary glands (data not shown). There were no overt changes in early morphogenesis during pregnancy, or during the first 5 days of pseudo-pregnancy (after mating with a vasectomized male; data not shown). In line with their normal virgin morphology, the mitotic indices of these bitransgenic glands were also normal (measured using Ki67 immunohistochemistry, or BrDU incorporation; data not shown). We conclude that ectopic Wnt4 expression is not sufficient to induce hyper side-branching.

### Wnt4 does not induce canonical Wnt signaling reporter gene expression in mammary epithelial and fibroblast cells in vitro

Recent studies suggest that the receptor species available determine the Wnt signaling responses, rather than any particular Wnt ligand [17,18]. Thus, Wnt ligands can induce so-called "canonical" Wnt signaling by forming a tertiary complex with Frizzled and LRP-5/6, while non-canonical Wnt signaling pathways are mediated through Frizzled alone or atypical receptor tyrosine kinases such as Ryk and Ror [19].

**Figure 4:**
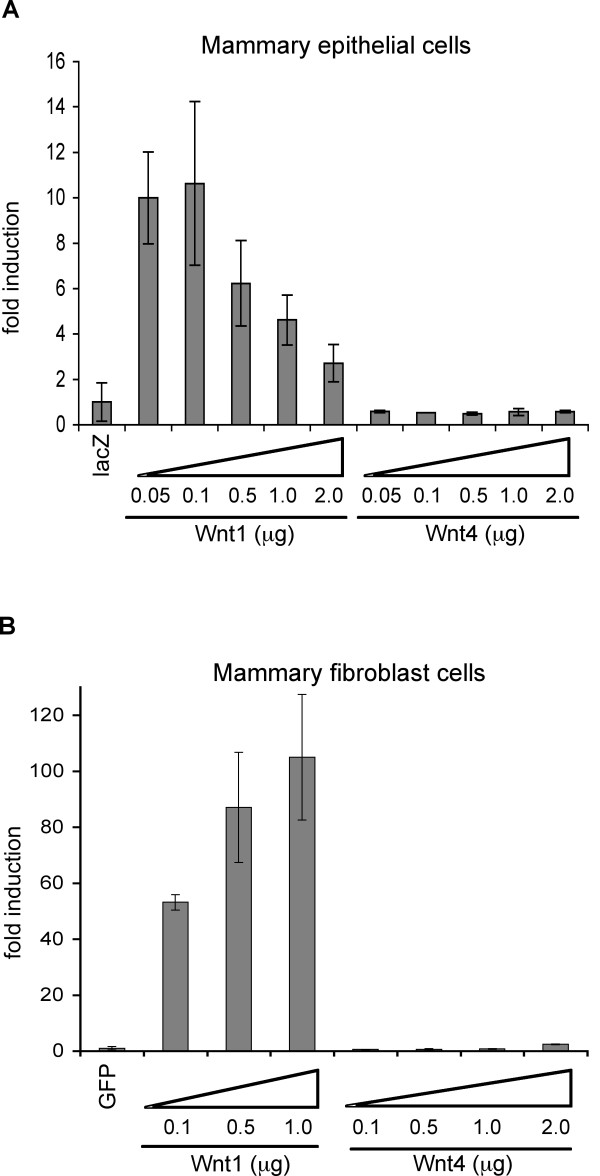
**Wnt4 does not induce canonical Wnt signaling in mammary epithelial or fibroblast cells *in vitro***. (A) Wnt reporter assay of primary mammary epithelial cells. Primary mammary epithelial cells were transfected with superTOP-flash reporter gene, a *Renilla *luciferase gene (a transfection control), and various amounts of Wnt4 expression vector (0.05~2.0 μg). Luciferase activity was measured 48 hrs after transfection, and results corrected for transfection efficiency (dividing by *Renilla *luciferase activity). Fold induction was calculated with respect to the negative control (*LacZ*-transfected cells). A Wnt1 expression vector was used as a positive control. (B) Wnt reporter assay of immortalized mammary fibroblast cells. Mammary fibroblast cells were prepared from FVB-Cdkn2a^-/- ^(*Ink4a/Afr *null) female mice as described in Materials and Methods, and superTOP-flash reporter assays were performed as above. Error bars = standard deviation of triplicate samples.

To evaluate whether mammary epithelial cells in culture could respond to Wnt4 with the activation of canonical Wnt signaling, we measured Wnt transactivation using a TCF-β catenin reporter assay. Primary mammary epithelial cells (prepared from adult virgin mammary glands) were transfected with Wnt ligand expression plasmids together with the superTOP-Flash reporter plasmid (encoding a luciferase gene downstream from eight TCF/LEF binding sites). Wnt1, a commonly used inducer of canonical Wnt signaling in mammary gland, was used as a positive control. Although ectopic Wnt1 expression induced reporter gene expression in mammary epithelial cells, Wnt4 did not (Fig. 4A). The specificity of this reporter gene assay was confirmed using a scrambled superFOP-flash reporter plasmid (data not shown).

Though it is known that the effects of Wnt4 are epithelial cell-autonomous (Brisken et al, 2000), the responder/effector cells do not necessarily have to be epithelial. Therefore, we tested the canonical response of mammary fibroblast cells (Fig. 4B). Again, we showed that reporter gene expression was easily induced by Wnt1 in a dose dependent manner, but not by Wnt4. Therefore, we conclude that Wnt4 is not a canonical effector in either cell context.

### Wnt4 is biologically active

The lack of functional activity associated with Wnt4 in these *in vivo *and *in vitro *analyses led us to examine whether the Wnt4 expression vector was generating biologically active protein. Zebrafish embryos have long been used to study the role of various Wnt ligands during development, and the expression pattern and functionality of Wnt ligands is remarkably highly conserved between mammals and zebrafish. Ectopic misexpression of zebrafish Wnt4 (zWnt4; zebrafish homolog of Wnt4) during embryogenesis has been characterized. At 26 hours, zWnt4 mRNA-injected embryos display phenotypes (such as cyclopia, notochord defects, misfolding or laterally displaced hindbrain, and shortened trunk and tail) that arise from an inhibition of convergence and extension movement [20].

**Figure 5:**
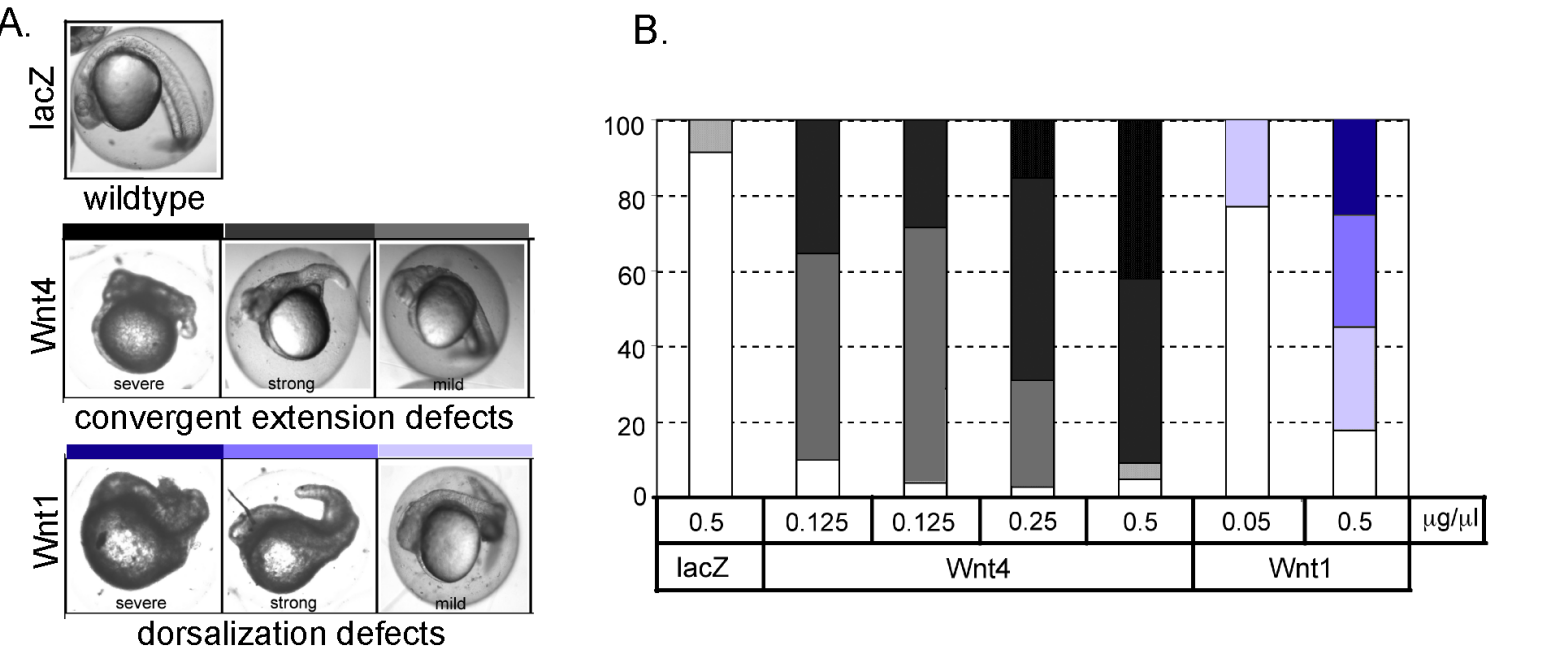
**Ectopic mouse Wnt4 expression induces a non-canonical Wnt phenotype during zebrafish development**. (A) Gain of function phenotypes induced by mouse Wnt4 in zebrafish embryo. Various doses of mouse Wnt4 or Wnt1 mRNA was injected to fertilized zebrafish eggs, and the phenotype was assessed 24 hrs after injection. (β-galactosidase (lacZ) mRNA was used as a negative control.) The phenotypes were classified into 3 groups (severe, strong and mild) based on the complexity of phenotype. (B) Wnt4 induces gain of function phenotypes in a dose dependent manner. On the y-axis, % total embryos are shown with each (color-coded) phenotype (severe to mild, shown in A), after injection with the indicated dose of control, Wnt1 or Wnt4 constructs.

Taking advantage of this, we injected mouse Wnt4 (mWnt4) mRNA (transcribed *in vitro*) into fertilized zebrafish embryos (1~4 cell stage) and examined the gain of function phenotypes after 24~26 hours. As predicted by the fact that mWnt4 and zWnt4 share over 80% identity in amino acid sequences, ectopic misexpression of mWnt4 displayed similar gain of function phenotypes to those previously reported zWnt4-induced phenotypes, including cyclopia, shortened tail and trunk, and abnormalities in brain (Fig. 5), thought to be the result of abnormal cell movement. Furthermore, phenotypes induced by mWnt4 were different from mWnt1. Although ectopic misexpression of mWnt1 also displayed developmental abnormalities in brain and shortened trunk and tail, mWnt1 induced unique dorsalization phenotypes such as "tail-up" and "bustled" [20]. We classified the gain of function phenotypes into 4 groups (severe, strong, mild and none), and quantified the phenotypes induced by different mRNA injection doses (Fig. 5). The quantification showed that both Wnt4 and Wnt1 induced the gain of function phenotypes in a dose dependent manner. Thus, we confirm that mouse Wnt4 is a biologically active protein that generates distinct phenotypes from Wnt1 (perhaps because of cell type-specific expression of Frizzled (or other) receptors).

### Wnt16 expression is induced by Wnt4 signaling

Our data show that although biologically active Wnt4 proteins are expressed in bitransgenic mammary glands, there is no significant development of lobuloalveoli. On the other hand, we confirmed that endogenous Wnt4 expression, associated with increased progesterone during the estrus cycle, correlates with transient side-branching development [10].

**Figure 6:**
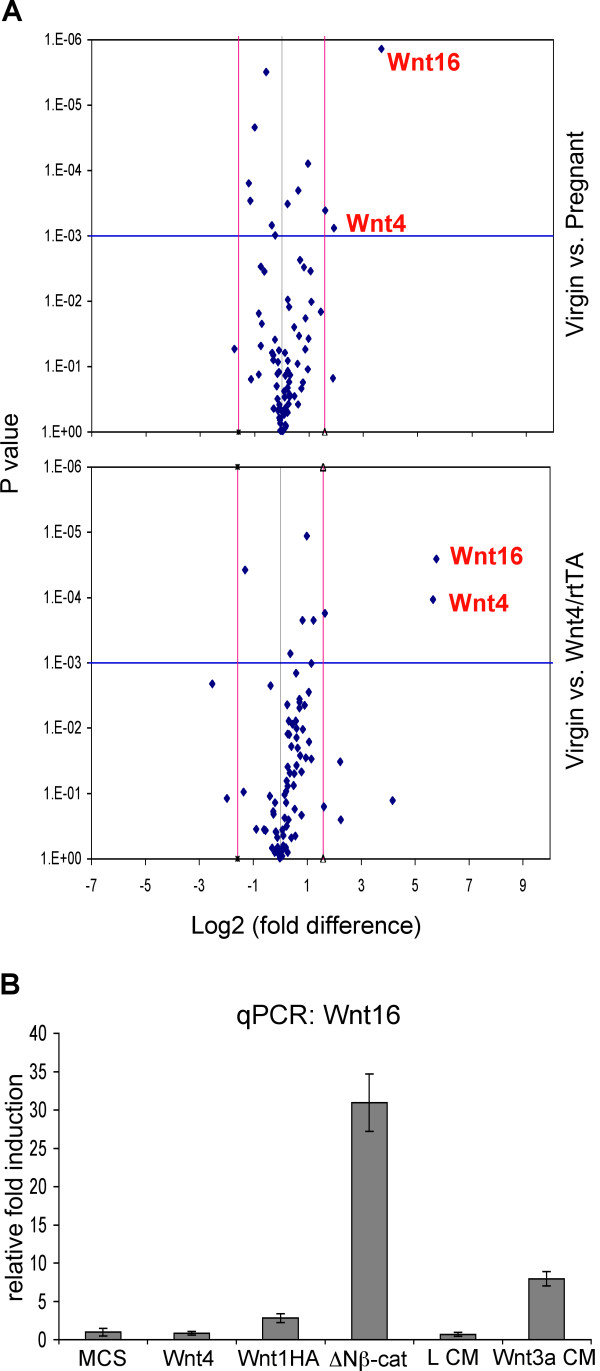
**Wnt16 expression is induced by Wnt4**. (A) Comparison of Wnt signaling-related gene expression profiles for virgin and pregnant (5.5 d.p) glands (upper panel) or for virgin and doxycycline-administered tet-Wnt4/MMTV-rtTA glands (lower panel). Vertical red lines indicate an arbitrary cut-off for significance (2 fold) up- or down-regulation, and the horizontal blue line denotes a significance p value of 0.001. (B) Wnt16 expression is induced by canonical Wnt signaling agonists in HC11 cells. HC11 cells were either transduced with Wnt signaling agonists encoding retroviruses, or were treated with conditioned media ± Wnt3a. Wnt16 mRNA amounts was measured using qPCR.

We presume that progesterone-regulated genes may include both a ligand and receptor pair. To test whether a cognate receptor (including the Frizzled (*Fzd*) proteins) was missing in virgin transgenic mice compared to pregnant glands, we assessed the expression levels of *Fzd *family genes using Wnt signaling-specific qPCR-based array (Superarray) analyses. The Wnt signaling specific array has 96 primer sets that include most Wnt signaling components and some housekeeping controls. We compared gene expression in early pregnant glands with that of transgenic doxycycline-administered tet-Wnt4/MMTV-rtTA glands and control virgin glands (Additional Files 1, 2, 3 &4). Only a few genes are significantly up- or down-regulated in early pregnant (5.5 d.p) glands or bitransgenic glands compared with virgin glands (Fig. 6A; summarized in Additional File 4). Prominent amongst these was Wnt4 (our positive control). Furthermore, contrary to our hypothesis, although one Fzd species, *Fzd9*, increased in pregnant glands, it increased more in Wnt4:rtTA transgenic glands, and there were no other significant and reproducible changes in other *Fzd *family genes (Fig. 6A and Additional Files 1, 2, 3 &4).

However, this analysis showed that the Wnt4 target gene, Wnt16 (described for endometrium) [21] was induced by 13-fold and 55-fold in pregnant and transgenic glands respectively (Fig. 6A; Additional File 4). We conclude that there is a Wnt4-binding receptor that signals a response in virgin gland.

In order to determine whether Wnt16 was a direct target of Wnt4 signaling in mammary epithelial cells, HC11 cells were either transduced with recombinant retroviruses encoding Wnt signaling agonists, or they were treated with conditioned medium from control (L cell) or Wnt3a- L cells, and total RNAs were prepared for qPCR analyses. Interestingly, Wnt16 expression was induced by canonical Wnt signaling effectors (Wnt1HA, ΔNβ-catenin (a constitutively active form of β-catenin), and Wnt3a) but not directly by Wnt4 (Fig. 6B). We tested whether co-addition of progesterone with Wnt4 would induce Wnt16 in HC11 cells, but it did not (data not shown). Notice that axin2, a robust Wnt signaling reporter [22], was not significantly induced in either the Wnt4:rtTA or pregnant glands (a result also shown by others). We cannot conclude whether Wnt16 is a canonical or non-canonical signaling target in mammary epithelial cells in vivo.

## Discussion

In order to study the published growth-associated activity of Wnt4 *in vivo *during mammary gland development, we generated bitransgenic mice that express Wnt4 in mammary glands in a doxycyline-inducible manner. Perhaps surprisingly, we show that despite high levels of biologically active Wnt4 in these transgenic mammary glands, there is no induction of lobuloalveoar side branching. We propose that Wnt4 may be necessary but not sufficient for morphogenesis.

### Wnt4 is not sufficient for lobuloalveolar development

A role for Wnt4 in lobuloalveolar development was first proposed by Bradbury et al. [23]. This study used recombinant retroviruses encoding Wnt4 under the control of β-actin promoter, to express Wnt4 ectopically in mammary epithelial cells in culture. When transplanted *in vivo *into fat pads, the transduced mammary epithelial cells developed highly branched ducts. However, only 18% of the reconstituted glands displayed the "pregnant-like" phenotype, while 55% of the reconstituted glands had normal virgin mammary gland structures. The authors proposed that this might be the result of a low infection efficiency or low dose of Wnt4 protein. It could be that retroviral transduction favors fibroblasts and endothelial cells, and that these cells modify the colonization pattern of mammary epithelial cells, without having a robust effect.

**Figure 7:**
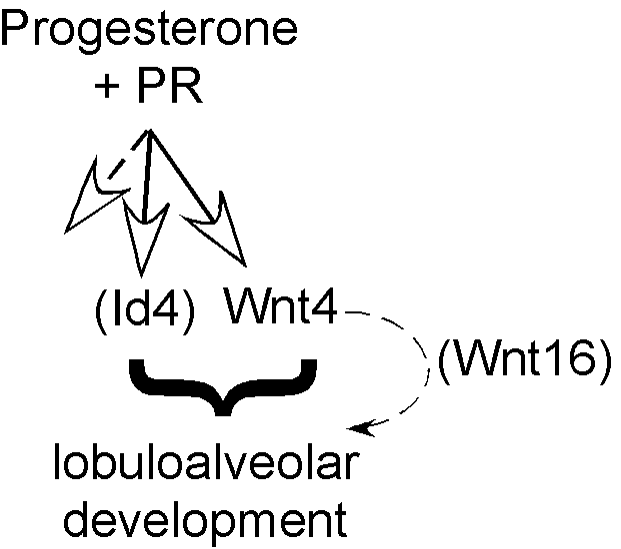
**Scheme of potential pathway for induction of lobuloalveolar development**. Accessory, progesterone-induced factors (besides Wnt4) may be required to promote growth.

Alternatively, it is possible that Wnt4 may induce ductal side-branching only in the presence of the correct endocrine environment. Thus, there is evidence that progesterone induces Wnt4 expression [10,24]. The hormone-induced program includes many different subprograms, and we propose that a cooperating factor is required for Wnt4-induced development. A good candidate as this cooperating factor is the b-HLH protein, Id4, one of the main factors induced in vivo by progesterone-exposure of PR-expressing luminal cells [25]. Loss of function of Id4 induces hypomorphic side-branching (Yi Li, personal communication), and gain of function induces hyperplasia (but not normal side-branching; John Lydon, personal communication). The requirement of (an)other progesterone-induced factor in addition to Wnt4 during lobuloalveolar development is shown in summary in Fig. 7. Another formal possibility to explain a lack of physiological response is that the relatively higher levels of Wnt4 (mRNA) we observe in transgenic glands (1.5× early pregnancy levels) is too high, or that the Wnt4 protein is unavailable to the responder cells (expressed predominantly in MMTV-positive luminal cells).s

To study the effect of loss of Wnt4 function, Brisken et al. transplanted mammary rudimentary epithelium isolated from *Wnt4 *null pups on the cleared fat pads (to rescue a mutations that is perinatal lethal because it causes kidney failure [26]. The *Wnt4 *null epithelium formed normal ductal structures in reconstituted virgin mammary glands, but the ductal side-branching was disrupted, specifically during early pregnancy [9], suggesting the essential function of Wnt4 in early lobuloalveolar development. Taken together, we propose that Wnt4 is required, but not sufficient for lobuloalveolar development during early pregnancy.

### Wnt4-induced signaling; Canonical or non-canonical?

Wnt4 has been classified as a non-canonical ligand. Thus, unlike canonical Wnt ligands such as Wnt1 and Wnt3a, Wnt4 was not able to transform C57 MG cells (an immortalized mouse mammary epithelial cell line of uncertain origin) *in vitro *[27], or stabilize endogenous β-catenin [28]. Consistent with those observations, we have shown that Wnt4 had no effect on superTOP-flash reporter gene expression in primary mammary epithelial cells and mammary fibroblast cells (Fig. 4). Moreover, injection of mouse Wnt4 to zebrafish embryos induced similar phenotypes to zWnt4, classified in the same functional group as Wnt5a, a prototype agonist of non-canonical Wnt signaling [20,29]. However, recent studies have shown that when provided with correct species of Frizzled and sufficient LRP protein, Wnt5a can act as a canonical agonist [17,18]. Indeed, Wnt4 activates canonical responses in MDCK kidney cells, and binds Fzd6, though this receptor is unlikely to be signaling receptor [30]. Receptor context is therefore key to the functional readout.

Often, Wnt4-dependent physiologies involve more than one cell type. For example, Wnt4 is required for tubulogenesis during kidney development (perhaps a similar epithelial process to ductal side-branch formation), and is induced in mesenchymal cells by an epithelial cell-derived signal [31]. Wnt4 expression induced the canonical reporter gene (TOP-gal) in mesenchymal cells [11], and ectopic β-catenin expression was sufficient to replace Wnt4-mediated signaling [32]. In a renal injury model, Wnt4 was able to stabilize endogenous β-catenin and induce reporter gene expression in certain myofibroblast cell lines [33,34]. Furthermore, Wnt4 activated canonical signaling in MDCK (Mardin-Darby Canine Kidney) cells [30]. However, other data suggests that Wnt4 inhibited canonical signaling by redirecting the subcellular localization of β catenin [35], or that it is key to hematopoiesis through a β catenin-independent activity [36]. These data show that although Wnt4 can function as a canonical signaling agonist in complex cell systems, it has other activities. Interestingly, expression of Wnt1 (a prototype agonist of canonical Wnt signaling) in mammary ductal epithelial cells through either MMTV-LTR promoter or recombinant retrovirus also causes hyper side-branching that cannot be recapitulated by expression of the downstream intracellular transactivator, β-catenin [23,37,38]. These observations support the idea that canonical signaling activation by soluble Wnt ligands in nearby mesenchymal cells through a paracrine mechanism is necessary for ductal side-branching morphogenesis [1].

Using qPCR-based array analyses, we found that Wnt16 expression is induced by Wnt4 *in vivo *(Fig. 6A). Furthermore, a recent study showed that expression of both Wnt4 and Wnt16 is regulated by PR and FOXO1 (forkhead transcription factor 1) during differentiation of endometrial stromal cells into decidual cells [21]. Thus, knockdown of FOXO1 using small interfering RNAs down-regulates Wnt4 and Wnt16 expression, and knockdown of PR disrupts the regulation of Wnt4 by FOXO1 in human endometrial stromal cells. These data indicate that Wnt16 is a downstream target gene of PR-Wnt4 signaling axis. Interestingly, however, our data also show that ectopic Wnt4-Wnt16 expression is not sufficient to induce side-branching development, suggesting that other cooperating factors need to be provided. Taken together, we propose that PR signaling activates not only Wnt4-Wnt16 expression, but also other cooperating factors, and the orchestrated crosstalk is required for lobuloalveolar development during early pregnancy (Fig. 7).

## Conclusion

In conclusion, we show that Wnt4 expression may be necessary, but is not sufficient, to induce lobuloalveolar development. We suggest that progesterone-induced collaborating factors may be required for Wnt4-mediated mammary gland morphogenesis.

## Methods

### Plasmid constructions

TMILA plasmid was kindly provided by Dr. Lewis A. Chodosh [15]. To generate the Wnt4 expression cassette, PCR amplified mouse Wnt4 cDNA (a gift of Dr. Trevor C. Dale, Cardiff University) was subcloned into the multiple cloning site of TMILA plasmid. Mouse Wnt4 and Wnt1 expression vectors were generated by inserting the cDNA clone downstream of an EF1α promoter sequences of a self-inactivating lentiviral vector (SIN-EF1α; a gift of Dr. Robert G. Hawley, American Red Cross). Canonical Wnt signaling reporter plasmids (superTOP-flash and superFOP-flash) were kindly provided by Drs. Ajamete Kaykas and Randall T. Moon (University of Washington). For *in vitro *transcription of mouse Wnt4 and Wnt1, cDNAs were subcloned into the multiple cloning site of the pCS2^+ ^plasmid that contains T7 and Sp6 promoter sequences.

### Mice

All mice used in this study were maintained on an inbred FVB/NJ background. They were housed and handled in accordance with NIH guidelines, and administered by the University of Wisconsin Animal Care and Use Committee. FVB-Cdkn2a^-/-^(Ink4a/Arf null) mice were obtained from MMHCC-NCI-Frederick. FVB-MMTV-rtTA mice (MTB line) were kindly provided by Dr. Lewis C. Chodosh [16]. To generate doxycycline inducible Wnt4 transgenic mice, the Wnt4 expression cassette (tet-Wnt4-IRES-luciferase) was cut out from TMILA-Wnt4 plasmid using *Not *I restriction enzyme, and the isolated fragment (~6.5 kb) was injected to fertilized oocytes from FVB/NJ background. Potential founders were identified by PCR-based genotyping using a luciferase gene-specific primer set (forward; 5'-ATG GAA GAC GCC AAA AAC-3', reverse; 5-'CTG AAA TCC CTG GTA ATC CG-3'). To generate tet-Wnt4/MMTV-rtTA bitransgenic mice, male tet-Wnt4 mice were mated with female MMTV-rtTA mice.

### Doxycycline administration

To induce transgene expression, transgenic mice were administered with 5% sucrose + 2 mg/ml doxycyline containing water. Doxycycline-containing water was changed every 2~3 days.

### Whole Mounts

Inguinal mammary glands were fixed in Acetic acid/Ethanol (1:3) solution overnight at 4°C with mixing, and stained in Carmine stain overnight. The stained glands were dehydrated through a series of washes in 70, 95, and 100% ethanol for 30 min each. Fat was dissolved in xylene. Pictures were taken by Axiovert 25 invert microscope with AxioCam color digital camera (Zeiss) using Open Lab software (Improvision).

### Cell preparation

To generate immortalized mammary fibroblast cells, endogenous epithelium-cleared mammary fat pads from 3 week old FVB-Cdkn2a^-/-^(Ink4a/Arf null) mice were chopped with scissors to ~1 mm^3 ^chucks and then digested with collagenase (3 mg/ml; Worthington) and trypsin (1.5 mg/ml; Worthington) for 90 min at 37°C with agitation. Enzyme digested tissues were washed with medium, and then filtered through a 40 μm cell strainer. The filter-through fraction was mostly single cells and these were plated on tissue culture plates. Only fibroblast cells grew out in DMEM+10% FBS medium, deduced from their expression of the fibroblast markers (Vimentin and SMAα), and their lack of epithelia marker expression (K8 and K5). Primary mammary epithelial cells were prepared as previously described [39]

### Luciferase assay

To measure luciferase reporter gene expression in doxycyline-treated mammary glands, tissues were homogenized in luciferase lysis buffer (Promega) using a glass douncer and pestle on ice. Insoluble tissue lysates were removed by centrifugation (12,000 g for 5 min), and their protein concentration was determined by micro BCA protein assay kit (Pierce). Luciferase activity was measured using 10 μg of protein lysate using Luciferase assay kit (Promega) and a Monolight 3010 luminometer (Analytical Luminescence Laboratory).

For the canonical Wnt signaling reporter (superTOP-flash) assay, 8 × 10^4 ^mammary epithelial cells or 5 × 10^4 ^mammary fibroblast cells were plated on 24-well plates, and cells were transfected with Wnt4 or Wnt1 expression vectors together with superTOP-flash reporter plasmid (0.1 μg) and renilla luciferase gene (0.01 μg) using LipofectAmine 2000 (Gibco BRL). Approximate transfection efficiencies were 10 and 30% for mammary epithelial cells and mammary fibroblasts respectively. The total transfected DNA amount was kept constant by adding either β-galactosidase or eGFP expression plasmid. Cells were harvested in 200 μl of passive luciferase lysis buffer (Promega) at 48 hrs after transfection, and both firefly and renilla luciferase activities were measured with 10 μl of cell lysate using Dual luciferase assay kit (Promega) as instructed by manufacturer. To normalize transfection efficiency, firefly luciferase activity was divided by the renilla luciferase activity. Fold induction was determined by dividing the luciferase unit results from samples transfected with Wnt expressing plasmids by the results from samples transfected with lacZ-containing control plasmids.

### Southern blot analysis

Genomic DNAs (gDNAs) were isolated from tail biopsies, and 10 μg of gDNA was cut with *EcoR *I restriction enzyme for 10 hrs at 37°C. Digested gDNA was precipitated with 100% EtOH and resuspended with 9 μl of H_2_O to separate through 0.8% agarose gel (TAE buffer, 25 V, overnight). Gels were rinsed with water and incubated in 0.25 M HCl solution for 30 min at room temperature (RT) with agitation, and then neutralized with 0.4 M NaOH (20 min at RT). DNAs were tranferred to Hybond N^+ ^membrane (Amersham Pharmacia Biotech) for 4 hrs using an alkaline capillary downward blotting method (0.4 M NaOH transfer buffer). Blotted membrane was hybridized with TMILA-Wnt4 specific probes (labeled by random priming of the TMILA-Wnt4 insert) using ULTRAhyb solutions (Ambion) as instructed by the manufacturer.

### Western blot analysis

Cells were lysed with lysis buffer (1% SDS + protease inhibitor cocktail (Roche) in H_2_O) and protein concentrations were determined by micro BCA protein assay kit. Equal amounts of total protein were separated using 10% SDS-PAGE, and electrotransferred to Immobilon P PVDF membrane (Millipore). Membrane was blocked with 10% non-fat milk + TBS-Tw (Tris buffered saline + 0.05% Tween-20) followed by incubation with anti-mouse Wnt4 antibody (1:500, clone 55010, rat monoclonal, R&D systems) for 1 hr at RT. Membranes were then incubated with HRP conjugated anti-rat IgG antibody (1:5000, Jackson ImmunoResearch Lab) for 1 hr at RT, and visualized using chemiluminescence reagent.

### Zebrafish injection

Capped Wnt4 (or Wnt1) mRNA was transcribed from pCS2^+^-Wnt4 (or Wnt1) plasmid using mMessage mMachine transcription kit (Ambion) according to the manufacturer's instructions. mRNA was suspended in 0.1% phenol red containing nuclease-free water and injected into wild type zebrafish embryos at 1~4 cell stage. The gain of function phenotype was assessed 24~26 hrs after injection.

### Real time quantitative PCR

Snap-frozen inguinal (lymph node removed) or thoracic mammary glands were homogenized and total RNA was isolated using ToTALLY RNA isolation kit (Ambion) according to the manufacturer's instruction. 4 μg of total RNA was reverse transcribed in 40 μl reaction volume using StrataScript reverse transcriptase (Stratagene). 1 μl of cDNA was mixed with 5 μl of Platinum Super Mix (Platinum SYBER Green qPCR SuperMix-UDG with Rox, Invitrogen) and 4 μl of forward and reverse primer (0.5 μM), and the PCR was performed using ABI7900-HT (50°C-2 min, 95°C-2 min, 45 cycles of 95°C-15 sec; 53°C-30 sec; 72°C-30 sec, and followed by a dissociation curve). The oligonucleotide sequences of primers are as follows: Wnt4-F; 5'-CGA GGA GTG CCA ATA CCA GT-3', Wnt4 R; 5'-GCC ACA CCT GCT GAA GAG AT-3', TBP-F; 5'-ACC TTA TGC TCA GGG CTT G-3', TBP-R; 5'-TGG TGT TCT GAA TAG GCT GTG-3', HPRT-F; 5'-CCT CAT GGA CTG ATT ATG GAC AG-3', HPRT-R; 5'-AAT CCA GCA GGT CAG CAA AG-3', K18-F; 5'-GAG GAG AGT ACC ACA GTT GTC-3', K18-R; CCC GAG GCT GTT CTC CAA G-3'. All data values were normalized by geomean of three different internal control genes (HPRT, TBP, and K18) and relative gene expression data were determined using the 2^-ΔΔCt ^method [40]; ΔCt_(sample) _= Ct_(sample) _- Ct_(geomean of internal control genes)_, ΔΔCt_(sample) _= ΔCt_(sample) _- ΔCt_(non-transgenic control sample)_. The relative fold induction was calculated by dividing each value by average value of non-transgenic control samples (7 week old gland).

### Superarray analysis

Total RNA isolation was performed using the RNeasy Lipid Tissue Mini Kit (Qiagen). Mammary gland tissue, weighing approximately 100 mg, was cut into smaller pieces and placed into 2 × 2 ml centrifuge tubes containing 1 ml of QIAzol Lysis Reagent. The tissues were homogenized (Polytron PT 2100), and RNA isolation was completed as per manufacturer's instructions.

Gene expression was quantified using the RT2 ProfilerTM PCR Array System (SABiosciences). 3.0 ug of total RNA (in 40 μl) from mammary gland was used for reverse transcription using the RT^2 ^First Strand Kit. Reactions were diluted to 13.5 ng/ul, RT2 Real-Time SYBER Green/ROX PCR Master Mix was mixed according to the manufacture's instructions. 10 μl reactions were loaded into each well of a 384 well plate, SABiosciences #APMM-043E-4 with 3 gene substitutions (Axin2, FZD9, FZD10), containing various primer pairs in quadruplicates. 4 samples were run on one plate, and then repeated to produce duplicate results. We used a two-step cycling program that was recommended by the manufacturer for use with the ABI 7900 HT. The Baseline and Threshold values were manually adjusted for each plate to the same settings (2-12 cycles for the Baseline, and 0.1 Threshold). Quality control for the data included: well volumes, dissociation curves, and fluorescence graphs were examined for problem wells; the array plates also contained controls for genomic contamination, reverse transcription, and PCR. Results were analyzed with Microsoft Excel spreadsheet templates found at .

## Abbreviations

Lrp5: Lipoprotein receptor related protein-5; PR: Progesterone receptor; K5: keratin 5; BrDU: Bromodeoxyuridine.

## Authors' contributions

YCK designed the experiments in consultation with CMA, and did the majority of the experimental work, together with the preparation and interpretation of results for publication, and prepared the manuscript. RJC designed and performed all the qPCR analyses. FJP consulted on how best to assay our Wnt4 expression constructs using Zebrafish, and how to interpret the phenotypes. CMA mentored the experimental design and execution, and edited and submitted the manuscript. All authors read and approved the final manuscript.

## Supplementary Material

Additional file 1 **Comparison of mRNA expressed by mammary glands from virgin and pregnant females**. qPCR assay of components involved in Wnt signaling (see Materials section; SuperArray from SABiosciences, with modifications), compared pairwise for two sample types; RNA extracted from mammary glands from mid-pregnant mice (5.5 days p.c) and from virgin mice (3- 4 months of age). Assay results are shown, together with the fold difference between the pairs, the statistical significance (shown in red where p ≤ 0.05) and the fold up- or down-regulation. The main differences (summarized in Fig. 6) are shown in pink (up-regulated) or blue (down-regulated). Click here for file

Additional file 2 **Comparison of mRNA expressed by mammary glands in Wnt4-expressing bitransgenic mice with mammary glands from control virgin females**. qPCR assay of components involved in Wnt signaling, compared as for File 1, for 3-4 month old mice (in the case of bitransgenic mice, exposed to doxycycline for 1 month). Click here for file

Additional file 3 **Comparison of mRNA expressed by mammary glands in Wnt4-expressing bitransgenic mice with mammary glands from control pregnant females**. qPCR assay of components involved in Wnt signaling, compared as for File 1, for mid-pregnant glands and 3-4 month old Wnt4 bitransgenic mice (exposed to doxycycline continuously). Click here for file

Additional file 4 **Summary of changes of gene expression in glands from Wnt4-expressing bitransgenic mice or pregnant mice, versus virgin glands**. Significant changes identified from Files 1-3 are summarized, to show the broad correspondence of changes between these two conditions (shown in yellow highlight). Click here for file
