# Supplementary material for: Wnt4 is not sufficient to induce lobuloalveolar mammary development
Source: BMC Dev Biol. 2009 Oct 30;9:55. doi: 10.1186/1471-213X-9-55 (PMC2777140; doi:10.1186/1471-213X-9-55)
Supplement: Additional file 1 — Comparison of mRNA expressed by mammary glands from virgin and pregnant females. qPCR assay of components involved in Wnt signaling (see Materials section; SuperArray from SABiosciences, with modifications), compared pairwise for two sample types; RNA extracted from mammary glands from mid-pregnant mice (5.5 days p.c) and from virgin mice (3- 4 months of age). Assay results are shown, together with the fold difference between the pairs, the statistical significance (shown in red where p ≤ 0.05) and the fold up- or down-regulation. The main differences (summarized in Fig. 6) are shown in pink (up-regulated) or blue (down-regulated). [file 1471-213X-9-55-S1.pdf]

Symbol	Well	AVG ΔC_t (Ct(GOI) - Ave Ct (HKG))		$2^{-\Delta C_t}$		Fold Difference	T-TEST	Fold Up- or Down-Regulation
		All Preg	Virgin Con	All Preg	Virgin Con	All Preg /Virgin Con	p value	All Preg /Virgin Con
Aes	A01	1.74	1.49	3.0E-01	3.6E-01	0.84	0.0010	-1.19
Apc	A02	3.77	3.62	7.3E-02	8.2E-02	0.90	0.3134	-1.12
Axin1	A03	6.26	6.35	1.3E-02	1.2E-02	1.06	0.2384	1.06
Bcl9	A04	5.51	6.12	2.2E-02	1.4E-02	1.53	0.0002	1.53
Btrc	A05	5.58	5.50	2.1E-02	2.2E-02	0.94	0.4352	-1.06
Ctnnbip1	A06	7.53	7.83	5.4E-03	4.4E-03	1.24	0.1355	1.24
Ccnd1	A07	2.70	3.55	1.5E-01	8.5E-02	1.81	0.0543	1.81
Ccnd2	A08	1.67	1.31	3.1E-01	4.0E-01	0.78	0.0007	-1.29
Ccnd3	A09	3.30	2.73	1.0E-01	1.5E-01	0.67	0.0000	-1.49
Csnk1a1	A10	1.39	3.27	3.8E-01	1.0E-01	3.68	0.1503	3.68
Csnk1d	A11	4.45	4.66	4.6E-02	3.9E-02	1.16	0.0003	1.16
Csnk2a1	A12	4.10	3.96	5.8E-02	6.4E-02	0.91	0.0856	-1.10
Ctbp1	B01	3.35	3.47	9.8E-02	9.0E-02	1.08	0.0611	1.08
Ctbp2	B02	3.63	4.08	8.1E-02	5.9E-02	1.37	0.0250	1.37
Ctnnb1	B03	1.78	1.89	2.9E-01	2.7E-01	1.08	0.2921	1.08
Daam1	B04	4.14	4.04	5.7E-02	6.1E-02	0.93	0.1211	-1.08
Dixdc1	B05	5.77	6.04	1.8E-02	1.5E-02	1.21	0.2630	1.21
Dkk1	B06	16.25	16.20	1.3E-05	1.3E-05	0.97	0.9504	-1.04
Dvl1	B07	16.11	15.27	1.4E-05	2.5E-05	0.56	0.0154	-1.80
Dvl2	B08	6.77	6.77	9.1E-03	9.2E-03	1.00	0.9485	-1.00
Ep300	B09	6.84	6.92	8.7E-03	8.3E-03	1.05	0.5328	1.05
Fbxw11	B10	4.16	4.01	5.6E-02	6.2E-02	0.90	0.1283	-1.11
Fbxw2	B11	3.47	3.42	9.0E-02	9.4E-02	0.97	0.4571	-1.04
Fbxw4	B12	5.24	5.30	2.6E-02	2.5E-02	1.04	0.4687	1.04
Fgf4	C01	14.71	12.96	3.7E-05	1.3E-04	0.30	0.0536	-3.35
Fosl1	C02	11.42	11.06	3.7E-04	4.7E-04	0.78	0.0613	-1.28
Foxn1	C03	17.48	17.76	5.5E-06	4.5E-06	1.22	0.2890	1.22
Frat1	C04	8.06	8.27	3.7E-03	3.2E-03	1.15	0.2107	1.15
Frzb	C05	17.38	16.54	5.9E-06	1.1E-05	0.56	0.1324	-1.80
Fshb	C06	17.48	17.69	5.5E-06	4.7E-06	1.16	0.5094	1.16
Fzd1	C07	4.52	4.22	4.4E-02	5.4E-02	0.81	0.0794	-1.24
Fzd2	C08	6.12	6.79	1.4E-02	9.0E-03	1.60	0.0024	1.60
Fzd3	C09	5.36	5.99	2.4E-02	1.6E-02	1.56	0.0338	1.56
Fzd4	C10	3.00	1.78	1.2E-01	2.9E-01	0.43	0.0002	-2.33
Fzd5	C11	8.02	8.12	3.8E-03	3.6E-03	1.07	0.5366	1.07
Fzd6	C12	5.60	5.81	2.1E-02	1.8E-02	1.16	0.0810	1.16
Fzd7	D01	6.61	6.79	1.0E-02	9.0E-03	1.14	0.4789	1.14
Fzd8	D02	14.73	14.86	3.7E-05	3.4E-05	1.09	0.8523	1.09
Gsk3b	D03	2.55	2.50	1.7E-01	1.8E-01	0.96	0.7345	-1.04
Jun	D04	4.24	4.11	5.3E-02	5.8E-02	0.91	0.4716	-1.10
Kremen1	D05	5.36	5.50	2.4E-02	2.2E-02	1.10	0.1362	1.10
Lef1	D06	13.12	12.82	1.1E-04	1.4E-04	0.81	0.4364	-1.23
Lrp5	D07	4.88	5.10	3.4E-02	2.9E-02	1.16	0.0095	1.16
Lrp6	D08	2.96	2.90	1.3E-01	1.3E-01	0.96	0.6243	-1.04
Myc	D09	2.48	3.36	1.8E-01	9.8E-02	1.83	0.0183	1.83
Nkd1	D10	9.19	8.06	1.7E-03	3.8E-03	0.46	0.1561	-2.20
Nlk	D11	5.48	5.22	2.2E-02	2.7E-02	0.84	0.0388	-1.19
Pitx2	D12	16.58	16.63	1.0E-05	9.9E-06	1.03	0.9658	1.03
Porcn	E01	6.69	6.51	9.7E-03	1.1E-02	0.88	0.2003	-1.14
Ppp2ca	E02	1.02	0.96	4.9E-01	5.1E-01	0.96	0.6570	-1.05
Ppp2r1a	E03	2.98	2.89	1.3E-01	1.3E-01	0.94	0.3834	-1.06
Ppp2r5d	E04	4.36	4.26	4.9E-02	5.2E-02	0.93	0.0561	-1.07
Pygo1	E05	6.49	6.63	1.1E-02	1.0E-02	1.10	0.4323	1.10
Rhou	E06	4.19	5.01	5.5E-02	3.1E-02	1.76	0.0030	1.76
Senp2	E07	4.75	4.66	3.7E-02	4.0E-02	0.94	0.6060	-1.07
Sfrp1	E08	5.10	5.32	2.9E-02	2.5E-02	1.17	0.1165	1.17
Sfrp2	E09	3.76	5.36	7.4E-02	2.4E-02	3.03	0.0004	3.03
Sfrp4	E10	4.81	4.04	3.6E-02	6.1E-02	0.59	0.0481	-1.71
Slc9a3r1	E11	4.31	5.36	5.1E-02	2.4E-02	2.08	0.0034	2.08
Sox17	E12	8.46	8.15	2.8E-03	3.5E-03	0.80	0.0667	-1.24
T	F01	16.50	17.09	1.1E-05	7.2E-06	1.51	0.3808	1.51
Tcf3	F02	10.09	9.09	9.2E-04	1.8E-03	0.50	0.0000	-2.00
Tcf7	F03	7.87	8.33	4.3E-03	3.1E-03	1.38	0.2816	1.38
Tle1	F04	5.50	5.77	2.2E-02	1.8E-02	1.20	0.0122	1.20
Tle2	F05	6.73	7.68	9.4E-03	4.9E-03	1.94	0.1096	1.94

This is another way to present the fold change.
If the fold change is positive, it means up-regulation.
If the fold change is negative, it means down-regulation.

Wif1	F06	5.29	6.73	2.5E-02	9.4E-03	**2.70**	**0.0145**	**2.70**
Wisp1	F07	10.03	11.12	9.5E-04	4.5E-04	**2.12**	**0.0103**	**2.12**
Wnt1	F08	12.31	11.57	2.0E-04	3.3E-04	0.60	**0.0221**	-1.66
Wnt10a	F09	10.56	10.58	6.6E-04	6.5E-04	1.02	0.9476	1.02
Wnt11	F10	7.92	7.15	4.1E-03	7.0E-03	0.59	**0.0029**	-1.71
Wnt16	F11	9.56	13.23	1.3E-03	1.0E-04	**12.73**	**0.0000**	**12.73**
Wnt2	F12	8.13	7.49	3.6E-03	5.6E-03	0.64	**0.0035**	-1.56
Wnt2b	G01	12.49	13.07	1.7E-04	1.2E-04	1.49	0.0901	1.49
Wnt3	G02	17.35	17.49	6.0E-06	5.4E-06	1.10	0.7877	1.10
Wnt3a	G03	17.52	17.76	5.3E-06	4.5E-06	1.19	0.3727	1.19
Wnt4	G04	6.41	8.33	1.2E-02	3.1E-03	**3.78**	**0.0008**	**3.78**
Wnt5a	G05	7.60	7.53	5.1E-03	5.4E-03	0.95	0.6149	-1.05
Wnt5b	G06	5.62	6.59	2.0E-02	1.0E-02	1.95	**0.0001**	1.95
Wnt6	G07	9.54	10.52	1.3E-03	6.8E-04	1.98	**0.0376**	1.98
Wnt7a	G08	16.99	17.76	7.7E-06	4.5E-06	1.71	0.1734	1.71
Wnt7b	G09	10.99	9.83	4.9E-04	1.1E-03	0.45	**0.0003**	**-2.24**
Wnt8a	G10	15.88	16.60	1.7E-05	1.0E-05	1.65	0.2161	1.65
Wnt8b	G11	14.63	14.78	3.9E-05	3.6E-05	1.10	0.8149	1.10
Wnt9a	G12	10.39	10.65	7.5E-04	6.2E-04	1.20	0.1732	1.20
Gusb	H01	3.47	3.94	9.0E-02	6.5E-02	1.39	**0.0001**	1.39
Hprt1	H02	1.15	0.77	4.5E-01	5.9E-01	0.77	**0.0000**	-1.30
Hsp90ab1	H03	-1.30	-0.79	2.5E+00	1.7E+00	1.43	**0.0000**	1.43
Gapdh	H04	-1.35	-1.55	2.5E+00	2.9E+00	0.87	0.2219	-1.16
Actb	H05	-1.97	-2.37	3.9E+00	5.2E+00	0.76	**0.0001**	-1.32
Axin2	H05	3.72	3.71	7.6E-02	7.6E-02	1.00	0.9928	-1.00
Fzd9	H05	12.79	14.55	1.4E-04	4.2E-05	**3.39**	**0.0128**	**3.39**
Fzd10	H05	10.27	10.17	8.1E-04	8.7E-04	0.94	0.5648	-1.07
