# Supplementary material for: Wnt4 is not sufficient to induce lobuloalveolar mammary development
Source: BMC Dev Biol. 2009 Oct 30;9:55. doi: 10.1186/1471-213X-9-55 (PMC2777140; doi:10.1186/1471-213X-9-55)
Supplement: Additional file 2 — Comparison of mRNA expressed by mammary glands in Wnt4-expressing bitransgenic mice with mammary glands from control virgin females. qPCR assay of components involved in Wnt signaling, compared as for File 1, for 3-4 month old mice (in the case of bitransgenic mice, exposed to doxycycline for 1 month). [file 1471-213X-9-55-S2.pdf]

Symbol	Well	AVG ΔC_t (Ct(GOI) - Ave Ct (HKG))		$2^{-\Delta C_t}$		Fold Difference	T-TEST	Fold Up- or Down-Regulation
		Wnt4	Virgin Con	Wnt4	Virgin Con	Wnt4 /Virgin Con	p value	Wnt4 /Virgin Con
Aes	A01	1.26	1.49	4.2E-01	3.6E-01	1.18	0.0647	1.18
Apc	A02	3.04	3.62	1.2E-01	8.2E-02	1.49	**0.0078**	1.49
Axin1	A03	6.09	6.35	1.5E-02	1.2E-02	1.19	**0.0044**	1.19
Bcl9	A04	5.85	6.12	1.7E-02	1.4E-02	1.21	0.0770	1.21
Btrc	A05	5.13	5.50	2.9E-02	2.2E-02	1.29	**0.0007**	1.29
Ctnnbip1	A06	7.60	7.83	5.2E-03	4.4E-03	1.18	0.3202	1.18
Ccnd1	A07	4.16	3.55	5.6E-02	8.5E-02	0.66	0.3583	-1.52
Ccnd2	A08	1.67	1.31	3.1E-01	4.0E-01	0.78	**0.0022**	-1.29
Ccnd3	A09	3.00	2.73	1.3E-01	1.5E-01	0.83	0.1889	-1.20
Csnk1a1	A10	1.02	3.27	4.9E-01	1.0E-01	4.74	0.2532	4.74
Csnk1d	A11	4.39	4.66	4.8E-02	3.9E-02	1.21	**0.0122**	1.21
Csnk2a1	A12	3.74	3.96	7.5E-02	6.4E-02	1.17	0.0924	1.17
Ctbp1	B01	3.31	3.47	1.0E-01	9.0E-02	1.12	0.1039	1.12
Ctbp2	B02	4.05	4.08	6.0E-02	5.9E-02	1.02	0.8985	1.02
Ctnnb1	B03	2.00	1.89	2.5E-01	2.7E-01	0.92	0.4708	-1.08
Daam1	B04	3.70	4.04	7.7E-02	6.1E-02	1.26	**0.0125**	1.26
Dixdc1	B05	4.98	6.04	3.2E-02	1.5E-02	2.09	**0.0163**	2.09
Dkk1	B06	16.22	16.20	1.3E-05	1.3E-05	0.99	0.9856	-1.01
Dvl1	B07	14.48	15.27	4.4E-05	2.5E-05	1.73	0.2168	1.73
Dvl2	B08	6.25	6.77	1.3E-02	9.2E-03	1.43	**0.0497**	1.43
Ep300	B09	6.21	6.92	1.4E-02	8.3E-03	1.64	**0.0040**	1.64
Fbxw11	B10	4.40	4.01	4.7E-02	6.2E-02	0.76	0.1100	-1.31
Fbxw2	B11	3.31	3.42	1.0E-01	9.4E-02	1.08	0.4405	1.08
Fbxw4	B12	4.48	5.30	4.5E-02	2.5E-02	1.77	**0.0002**	1.77
Fgf4	C01	12.55	12.96	1.7E-04	1.3E-04	1.33	0.4789	1.33
Fosl1	C02	10.16	11.06	8.7E-04	4.7E-04	1.86	**0.0045**	1.86
Foxn1	C03	16.54	17.76	1.1E-05	4.5E-06	2.34	**0.0002**	2.34
Frat1	C04	7.62	8.27	5.1E-03	3.2E-03	1.57	**0.0200**	1.57
Frzb	C05	14.92	16.54	3.2E-05	1.1E-05	3.06	0.1606	3.06
Fshb	C06	16.54	17.69	1.1E-05	4.7E-06	2.22	**0.0010**	2.22
Fzd1	C07	4.31	4.22	5.0E-02	5.4E-02	0.94	0.6698	-1.07
Fzd2	C08	6.96	6.79	8.0E-03	9.0E-03	0.89	0.3883	-1.13
Fzd3	C09	5.39	5.99	2.4E-02	1.6E-02	1.52	**0.0377**	1.52
Fzd4	C10	1.48	1.78	3.6E-01	2.9E-01	1.23	0.2521	1.23
Fzd5	C11	7.29	8.12	6.4E-03	3.6E-03	1.78	**0.0105**	1.78
Fzd6	C12	5.46	5.81	2.3E-02	1.8E-02	1.28	**0.0484**	1.28
Fzd7	D01	6.08	6.79	1.5E-02	9.0E-03	1.64	**0.0049**	1.64
Fzd8	D02	14.33	14.86	4.9E-05	3.4E-05	1.45	0.1722	1.45
Gsk3b	D03	2.33	2.50	2.0E-01	1.8E-01	1.12	0.2376	1.12
Jun	D04	3.36	4.11	9.7E-02	5.8E-02	1.67	**0.0263**	1.67
Kremen1	D05	4.91	5.50	3.3E-02	2.2E-02	1.50	**0.0014**	1.50
Lef1	D06	11.76	12.82	2.9E-04	1.4E-04	2.08	**0.0028**	2.08
Lrp5	D07	4.12	5.10	5.7E-02	2.9E-02	1.97	**0.0000**	1.97
Lrp6	D08	2.30	2.90	2.0E-01	1.3E-01	1.52	**0.0101**	1.52
Myc	D09	3.15	3.36	1.1E-01	9.8E-02	1.16	0.6769	1.16
Nkd1	D10	7.78	8.06	4.5E-03	3.8E-03	1.21	0.7985	1.21
Nlk	D11	5.00	5.22	3.1E-02	2.7E-02	1.17	0.1381	1.17
Pitx2	D12	12.47	16.63	1.8E-04	9.9E-06	17.88	0.1278	17.88
Porcn	E01	6.43	6.51	1.2E-02	1.1E-02	1.06	0.3655	1.06
Ppp2ca	E02	1.11	0.96	4.6E-01	5.1E-01	0.90	0.3938	-1.11
Ppp2r1a	E03	3.08	2.89	1.2E-01	1.3E-01	0.88	0.1383	-1.14
Ppp2r5d	E04	4.28	4.26	5.2E-02	5.2E-02	0.99	0.9009	-1.01
Pygo1	E05	6.18	6.63	1.4E-02	1.0E-02	1.37	**0.0088**	1.37
Rhou	E06	4.30	5.01	5.1E-02	3.1E-02	1.63	**0.0036**	1.63
Senp2	E07	4.34	4.66	4.9E-02	4.0E-02	1.24	**0.0077**	1.24
Sfrp1	E08	4.82	5.32	3.5E-02	2.5E-02	1.42	0.0758	1.42
Sfrp2	E09	5.25	5.36	2.6E-02	2.4E-02	1.08	0.6375	1.08
Sfrp4	E10	6.55	4.04	1.1E-02	6.1E-02	0.17	**0.0021**	-5.72
Slc9a3r1	E11	5.47	5.36	2.3E-02	2.4E-02	0.93	0.7985	-1.08
Sox17	E12	7.55	8.15	5.4E-03	3.5E-03	1.52	**0.0139**	1.52
T	F01	16.54	17.09	1.1E-05	7.2E-06	1.47	0.4443	1.47
Tcf3	F02	9.00	9.09	2.0E-03	1.8E-03	1.07	0.7223	1.07
Tcf7	F03	8.87	8.33	2.1E-03	3.1E-03	0.69	0.3681	-1.46
Tle1	F04	5.35	5.77	2.4E-02	1.8E-02	1.33	**0.0190**	1.33
Tle2	F05	5.46	7.68	2.3E-02	4.9E-03	4.67	**0.0328**	4.67

This is another way to present the fold change.
If the fold change is positive, it means up-regulation.
If the fold change is negative, it means down-regulation.

Wif1	F06	7.03	6.73	7.7E-03	9.4E-03	0.81	0.6869	-1.23
Wisp1	F07	9.97	11.12	1.0E-03	4.5E-04	**2.22**	**0.0295**	**2.22**
Wnt1	F08	13.56	11.57	8.3E-05	3.3E-04	**0.25**	0.1186	**-3.95**
Wnt10a	F09	11.95	10.58	2.5E-04	6.5E-04	0.39	0.0952	**-2.57**
Wnt11	F10	7.41	7.15	5.9E-03	7.0E-03	0.84	0.2085	-1.19
Wnt16	F11	7.44	13.23	5.8E-03	1.0E-04	**55.41**	**0.0000**	**55.41**
Wnt2	F12	8.79	7.49	2.3E-03	5.6E-03	0.41	**0.0000**	**-2.46**
Wnt2b	G01	13.96	13.07	6.3E-05	1.2E-04	0.54	0.3556	-1.86
Wnt3	G02	16.54	17.49	1.1E-05	5.4E-06	1.94	**0.0284**	1.94
Wnt3a	G03	16.54	17.76	1.1E-05	4.5E-06	**2.34**	**0.0002**	**2.34**
Wnt4	G04	2.67	8.33	1.6E-01	3.1E-03	**50.72**	**0.0001**	**50.72**
Wnt5a	G05	7.46	7.53	5.7E-03	5.4E-03	1.05	0.7277	1.05
Wnt5b	G06	4.94	6.59	3.3E-02	1.0E-02	**3.14**	**0.0002**	**3.14**
Wnt6	G07	10.63	10.52	6.3E-04	6.8E-04	0.93	0.8174	-1.07
Wnt7a	G08	16.54	17.76	1.1E-05	4.5E-06	**2.34**	**0.0002**	**2.34**
Wnt7b	G09	9.55	9.83	1.3E-03	1.1E-03	1.21	**0.0396**	1.21
Wnt8a	G10	16.54	16.60	1.1E-05	1.0E-05	1.04	0.9143	1.04
Wnt8b	G11	14.98	14.78	3.1E-05	3.6E-05	0.87	0.8059	-1.15
Wnt9a	G12	9.87	10.65	1.1E-03	6.2E-04	1.72	**0.0467**	1.72
Gusb	H01	4.25	3.94	5.2E-02	6.5E-02	0.80	0.0993	-1.24
Hprt1	H02	0.64	0.77	6.4E-01	5.9E-01	1.09	0.3994	1.09
Hsp90ab1	H03	-1.30	-0.79	2.5E+00	1.7E+00	1.43	**0.0002**	1.43
Gapdh	H04	-2.35	-1.55	5.1E+00	2.9E+00	1.74	0.0776	1.74
Actb	H05	-1.24	-2.37	2.4E+00	5.2E+00	0.46	**0.0000**	**-2.19**
Axin2	H08	2.86	3.71	1.4E-01	7.6E-02	1.80	0.1045	1.80
Fzd9	H09	11.43	14.55	3.6E-04	4.2E-05	**8.70**	**0.0155**	**8.70**
Fzd10	H10	10.78	10.17	5.7E-04	8.7E-04	0.65	0.1863	-1.53
