# Supplementary material for: Wnt4 is not sufficient to induce lobuloalveolar mammary development
Source: BMC Dev Biol. 2009 Oct 30;9:55. doi: 10.1186/1471-213X-9-55 (PMC2777140; doi:10.1186/1471-213X-9-55)
Supplement: Additional file 3 — Comparison of mRNA expressed by mammary glands in Wnt4-expressing bitransgenic mice with mammary glands from control pregnant females. qPCR assay of components involved in Wnt signaling, compared as for File 1, for mid-pregnant glands and 3-4 month old Wnt4 bitransgenic mice (exposed to doxycycline continuously). [file 1471-213X-9-55-S3.pdf]

Symbol	Well	AVG ΔC_t (Ct(GOI) - Ave Ct (HKG))		$2^{\wedge}-\Delta C_t$		Fold Difference	T-TEST	Fold Up- or Down-Regulation
		All Preg	Wnt4	All Preg	Wnt4	All Preg /Wnt4	p value	All Preg /Wnt4
Aes	A01	1.74	1.26	3.0E-01	4.2E-01	0.72	0.0002	-1.40
Apc	A02	3.77	3.04	7.3E-02	1.2E-01	0.60	0.0020	-1.66
Axin1	A03	6.26	6.09	1.3E-02	1.5E-02	0.89	0.0418	-1.12
Bcl9	A04	5.51	5.85	2.2E-02	1.7E-02	1.26	0.0231	1.26
Btrc	A05	5.58	5.13	2.1E-02	2.9E-02	0.73	0.0011	-1.37
Ctnnbip1	A06	7.53	7.60	5.4E-03	5.2E-03	1.05	0.7410	1.05
Ccnd1	A07	2.70	4.16	1.5E-01	5.6E-02	2.75	0.0000	2.75
Ccnd2	A08	1.67	1.67	3.1E-01	3.1E-01	1.00	0.9915	1.00
Ccnd3	A09	3.30	3.00	1.0E-01	1.3E-01	0.81	0.0401	-1.24
Csnk1a1	A10	1.39	1.02	3.8E-01	4.9E-01	0.78	0.0986	-1.29
Csnk1d	A11	4.45	4.39	4.6E-02	4.8E-02	0.96	0.2888	-1.05
Csnk2a1	A12	4.10	3.74	5.8E-02	7.5E-02	0.78	0.0004	-1.28
Ctbp1	B01	3.35	3.31	9.8E-02	1.0E-01	0.97	0.2407	-1.03
Ctbp2	B02	3.63	4.05	8.1E-02	6.0E-02	1.34	0.0224	1.34
Ctnnb1	B03	1.78	2.00	2.9E-01	2.5E-01	1.16	0.0176	1.16
Daam1	B04	4.14	3.70	5.7E-02	7.7E-02	0.74	0.0002	-1.35
Dixdc1	B05	5.77	4.98	1.8E-02	3.2E-02	0.58	0.0001	-1.72
Dkk1	B06	16.25	16.22	1.3E-05	1.3E-05	0.98	0.9535	-1.02
Dvl1	B07	16.11	14.48	1.4E-05	4.4E-05	0.32	0.0047	-3.10
Dvl2	B08	6.77	6.25	9.1E-03	1.3E-02	0.70	0.0091	-1.44
Ep300	B09	6.84	6.21	8.7E-03	1.4E-02	0.64	0.0001	-1.55
Fbxw11	B10	4.16	4.40	5.6E-02	4.7E-02	1.18	0.0931	1.18
Fbxw2	B11	3.47	3.31	9.0E-02	1.0E-01	0.89	0.1202	-1.12
Fbxw4	B12	5.24	4.48	2.6E-02	4.5E-02	0.59	0.0000	-1.69
Fgf4	C01	14.71	12.55	3.7E-05	1.7E-04	0.22	0.0166	-4.46
Fosl1	C02	11.42	10.16	3.7E-04	8.7E-04	0.42	0.0000	-2.39
Foxn1	C03	17.48	16.54	5.5E-06	1.1E-05	0.52	0.0057	-1.92
Frat1	C04	8.06	7.62	3.7E-03	5.1E-03	0.74	0.0008	-1.36
Frzb	C05	17.38	14.92	5.9E-06	3.2E-05	0.18	0.0023	-5.49
Fshb	C06	17.48	16.54	5.5E-06	1.1E-05	0.52	0.0123	-1.92
Fzd1	C07	4.52	4.31	4.4E-02	5.0E-02	0.86	0.0972	-1.16
Fzd2	C08	6.12	6.96	1.4E-02	8.0E-03	1.80	0.0000	1.80
Fzd3	C09	5.36	5.39	2.4E-02	2.4E-02	1.03	0.8864	1.03
Fzd4	C10	3.00	1.48	1.2E-01	3.6E-01	0.35	0.0000	-2.87
Fzd5	C11	8.02	7.29	3.8E-03	6.4E-03	0.60	0.0051	-1.66
Fzd6	C12	5.60	5.46	2.1E-02	2.3E-02	0.91	0.2256	-1.10
Fzd7	D01	6.61	6.08	1.0E-02	1.5E-02	0.69	0.0545	-1.44
Fzd8	D02	14.73	14.33	3.7E-05	4.9E-05	0.75	0.5351	-1.32
Gsk3b	D03	2.55	2.33	1.7E-01	2.0E-01	0.86	0.2027	-1.16
Jun	D04	4.24	3.36	5.3E-02	9.7E-02	0.54	0.0000	-1.84
Kremen1	D05	5.36	4.91	2.4E-02	3.3E-02	0.73	0.0007	-1.36
Lef1	D06	13.12	11.76	1.1E-04	2.9E-04	0.39	0.0046	-2.57
Lrp5	D07	4.88	4.12	3.4E-02	5.7E-02	0.59	0.0000	-1.69
Lrp6	D08	2.96	2.30	1.3E-01	2.0E-01	0.63	0.0001	-1.58
Myc	D09	2.48	3.15	1.8E-01	1.1E-01	1.58	0.0028	1.58
Nkd1	D10	9.19	7.78	1.7E-03	4.5E-03	0.38	0.0525	-2.66
Nlk	D11	5.48	5.00	2.2E-02	3.1E-02	0.72	0.0019	-1.39
Pitx2	D12	16.58	12.47	1.0E-05	1.8E-04	0.06	0.0351	-17.35
Porcn	E01	6.69	6.43	9.7E-03	1.2E-02	0.83	0.0858	-1.20
Ppp2ca	E02	1.02	1.11	4.9E-01	4.6E-01	1.06	0.5986	1.06
Ppp2r1a	E03	2.98	3.08	1.3E-01	1.2E-01	1.07	0.3488	1.07
Ppp2r5d	E04	4.36	4.28	4.9E-02	5.2E-02	0.94	0.3205	-1.06
Pygo1	E05	6.49	6.18	1.1E-02	1.4E-02	0.80	0.0534	-1.25
Rhou	E06	4.19	4.30	5.5E-02	5.1E-02	1.08	0.5889	1.08
Senp2	E07	4.75	4.34	3.7E-02	4.9E-02	0.75	0.0466	-1.32
Sfrp1	E08	5.10	4.82	2.9E-02	3.5E-02	0.82	0.0424	-1.21
Sfrp2	E09	3.76	5.25	7.4E-02	2.6E-02	2.81	0.0003	2.81
Sfrp4	E10	4.81	6.55	3.6E-02	1.1E-02	3.35	0.0000	3.35
Slc9a3r1	E11	4.31	5.47	5.1E-02	2.3E-02	2.24	0.0000	2.24
Sox17	E12	8.46	7.55	2.8E-03	5.4E-03	0.53	0.0002	-1.88
T	F01	16.50	16.54	1.1E-05	1.1E-05	1.03	0.9383	1.03
Tcf3	F02	10.09	9.00	9.2E-04	2.0E-03	0.47	0.0002	-2.13
Tcf7	F03	7.87	8.87	4.3E-03	2.1E-03	2.00	0.0011	2.00
Tle1	F04	5.50	5.35	2.2E-02	2.4E-02	0.90	0.1662	-1.11
Tle2	F05	6.73	5.46	9.4E-03	2.3E-02	0.41	0.0000	-2.41

This is another way to present the fold change.
If the fold change is positive, it means up-regulation.
If the fold change is negative, it means down-regulation.

Wif1	F06	5.29	7.03	2.5E-02	7.7E-03	**3.33**	**0.0000**	**3.33**
Wisp1	F07	10.03	9.97	9.5E-04	1.0E-03	0.96	0.8099	-1.05
Wnt1	F08	12.31	13.56	2.0E-04	8.3E-05	**2.38**	0.1374	**2.38**
Wnt10a	F09	10.56	11.95	6.6E-04	2.5E-04	**2.62**	**0.0009**	**2.62**
Wnt11	F10	7.92	7.41	4.1E-03	5.9E-03	0.70	**0.0223**	-1.43
Wnt16	F11	9.56	7.44	1.3E-03	5.8E-03	**0.23**	**0.0000**	**-4.35**
Wnt2	F12	8.13	8.79	3.6E-03	2.3E-03	1.58	**0.0019**	1.58
Wnt2b	G01	12.49	13.96	1.7E-04	6.3E-05	**2.76**	**0.0255**	**2.76**
Wnt3	G02	17.35	16.54	6.0E-06	1.1E-05	0.57	0.1055	-1.76
Wnt3a	G03	17.52	16.54	5.3E-06	1.1E-05	0.51	**0.0054**	-1.98
Wnt4	G04	6.41	2.67	1.2E-02	1.6E-01	**0.07**	**0.0000**	**-13.41**
Wnt5a	G05	7.60	7.46	5.1E-03	5.7E-03	0.90	0.4580	-1.11
Wnt5b	G06	5.62	4.94	2.0E-02	3.3E-02	0.62	**0.0005**	-1.61
Wnt6	G07	9.54	10.63	1.3E-03	6.3E-04	**2.12**	**0.0102**	**2.12**
Wnt7a	G08	16.99	16.54	7.7E-06	1.1E-05	0.73	0.4175	-1.37
Wnt7b	G09	10.99	9.55	4.9E-04	1.3E-03	0.37	**0.0001**	**-2.71**
Wnt8a	G10	15.88	16.54	1.7E-05	1.1E-05	1.58	0.1531	1.58
Wnt8b	G11	14.63	14.98	3.9E-05	3.1E-05	1.27	0.6449	1.27
Wnt9a	G12	10.39	9.87	7.5E-04	1.1E-03	0.70	**0.0155**	-1.43
Gusb	H01	3.47	4.25	9.0E-02	5.2E-02	1.73	**0.0001**	1.73
Hprt1	H02	1.15	0.64	4.5E-01	6.4E-01	0.70	**0.0004**	-1.42
Hsp90ab1	H03	-1.30	-1.30	2.5E+00	2.5E+00	1.00	0.9865	-1.00
Gapdh	H04	-1.35	-2.35	2.5E+00	5.1E+00	0.50	**0.0042**	**-2.01**
Actb	H05	-1.97	-1.24	3.9E+00	2.4E+00	1.66	**0.0000**	1.66
Axin2	H05	3.72	2.86	7.6E-02	1.4E-01	0.55	**0.0221**	-1.80
Fzd9	H05	12.79	11.43	1.4E-04	3.6E-04	0.39	**0.0315**	**-2.56**
Fzd10	H05	10.27	10.78	8.1E-04	5.7E-04	1.43	0.0959	1.43
