# Supplementary material for: Wnt4 is not sufficient to induce lobuloalveolar mammary development
Source: BMC Dev Biol. 2009 Oct 30;9:55. doi: 10.1186/1471-213X-9-55 (PMC2777140; doi:10.1186/1471-213X-9-55)
Supplement: Additional file 4 — Summary of changes of gene expression in glands from Wnt4-expressing bitransgenic mice or pregnant mice, versus virgin glands. Significant changes identified from Files 1-3 are summarized, to show the broad correspondence of changes between these two conditions (shown in yellow highlight). [file 1471-213X-9-55-S4.pdf]

Wnt4/rtTA		pregnant		Wnt4/rtTA		pregnant	
up	fold	up	fold	down	fold	down	fold
WISP1	2.2	WISP1	2.1	sFRP4	5.7	CK2	3.7
Fzd9	8.7	Fzd9	3.4	Wnt2	2.5	Fgf4	3.3
Wnt4	51	Wnt4	3.8	ActinB	2.2	Fzd4	2.3
Wnt16	55	Wnt16	12.7			Tcf3	2
Wnt5b	3.1	Wnt5b	2				
Tie2	4.7	sFRP2	3				
Wnt3a	2.3	WIF1	2.7				
Wnt7a	2.3	Wnt6	2				
		Myc	1.8				
